# Empowering large chemical knowledge bases for exposomics: PubChemLite meets MetFrag

**DOI:** 10.1186/s13321-021-00489-0

**Published:** 2021-03-08

**Authors:** Emma L. Schymanski, Todor Kondić, Steffen Neumann, Paul A. Thiessen, Jian Zhang, Evan E. Bolton

**Affiliations:** 1 Luxembourg Centre for Systems Biomedicine (LCSB), University of Luxembourg, 6 avenue du Swing, 4367 Belvaux, Luxembourg; 2 Bioinformatics and Scientific Data, Leibniz Institute of Plant Biochemistry (IPB Halle), 06120 Halle, Germany; 3 German Centre for Integrative Biodiversity Research (iDiv), Halle-Jena-Leipzig, Deutscher Platz 5e, 04103 Leipzig, Germany; 4 National Center for Biotechnology Information, National Library of Medicine, National Institutes of Health, Bethesda, MD 20894 USA

**Keywords:** Chemical database, Compound database, Compound knowledge base, Cheminformatics, High resolution mass spectrometry, Identification, Environmental science, Exposomics, FAIR, Open science

## Abstract

Compound (or chemical) databases are an invaluable resource for many scientific disciplines. Exposomics researchers need to find and identify relevant chemicals that cover the entirety of potential (chemical and other) exposures over entire lifetimes. This daunting task, with over 100 million chemicals in the largest chemical databases, coupled with broadly acknowledged knowledge gaps in these resources, leaves researchers faced with too much—yet not enough—information at the same time to perform comprehensive exposomics research. Furthermore, the improvements in analytical technologies and computational mass spectrometry workflows coupled with the rapid growth in databases and increasing demand for high throughput “big data” services from the research community present significant challenges for both data hosts and workflow developers. This article explores how to reduce candidate search spaces in non-target small molecule identification workflows, while increasing content usability in the context of environmental and exposomics analyses, so as to profit from the increasing size and information content of large compound databases, while increasing efficiency at the same time. In this article, these methods are explored using PubChem, the NORMAN Network Suspect List Exchange and the in silico fragmentation approach MetFrag. A subset of the PubChem database relevant for exposomics, PubChemLite, is presented as a database resource that can be (and has been) integrated into current workflows for high resolution mass spectrometry. Benchmarking datasets from earlier publications are used to show how experimental knowledge and existing datasets can be used to detect and fill gaps in compound databases to progressively improve large resources such as PubChem, and topic-specific subsets such as PubChemLite. PubChemLite is a living collection, updating as annotation content in PubChem is updated, and exported to allow direct integration into existing workflows such as MetFrag. The source code and files necessary to recreate or adjust this are jointly hosted between the research parties (see data availability statement). This effort shows that enhancing the FAIRness (Findability, Accessibility, Interoperability and Reusability) of open resources can mutually enhance several resources for whole community benefit. The authors explicitly welcome additional community input on ideas for future developments.

## Introduction

Compound (or chemical) databases are an invaluable resource for many scientific disciplines. Through the joint evolution over the last decade of high resolution mass spectrometry (HR-MS), cheminformatics techniques and openly available compound databases, a whole new world for identifying small molecules in complex samples has emerged. Despite many advances, chemical identification is still generally considered a bottleneck in many research fields (see e.g. [1, 2]). Interest in the exposome [3] and the related exposomics field has increased as awareness of the influence of the external environment on health and disease has increased [4]. Exposomics requires researchers to find and identify relevant chemicals that cover the entirety of potential (chemical and other) exposures over entire lifetimes [4–6], significantly adding to the identification challenge.

Scientific disciplines such as environmental science, metabolomics, forensics and exposomics are focusing increasingly on high throughput data exploration with high resolution mass spectrometry (HR-MS) techniques [4, 7, 8]. Mass spectral libraries, which can be used to obtain rapid tentative identifications of relatively high confidence [9–11] still only cover a fraction of chemical information resources relevant in exposomics [9], metabolomics [12] or in complex samples in general [13, 14]. This is especially true for HR-MS techniques, which are inherently limited by the availability of reference standards as well as the relative youth and lack of standardization in the field [9]. Alternative methods to annotate detected exact masses in HR-MS studies beyond spectral library searching began emerging around 2010 by searching compound (i.e., chemical) databases for possible candidates using the exact mass or calculated molecular formula, and ranking these using in silico techniques to sort candidates using the measured fragmentation information. The plethora of identification methods now available are described and compared in detail elsewhere [14–17]. A wide variety of (generally open) compound databases are typically used as information sources for these identification efforts, containing anything between tens to hundreds of thousands (e.g. KEGG [18], HMDB [19, 20], CompTox [21]) and tens of millions of structures (e.g. ChemSpider [22] and PubChem [23–25]). Most of these resources and, consequently, the number of candidates per exact mass/formula, are expanding significantly over time. Typical queries with smaller databases return tens to hundreds of candidates, whereas typical queries with large databases such as PubChem now return thousands to tens of thousands of candidates per exact mass/formula query. For instance, querying HMDB, CompTox and PubChem with the formula C_10_H_14_N_2_ via the MetFrag [26, 27] web interface (12 August 2020) returns 4, 225 and 3704 candidates, respectively.

A major challenge in correctly identifying a chemical based on exact mass (or formula) and fragmentation information alone arises due to the relatively little information conveyed in the fragmentation spectrum. During one open community evaluation approach, the 2016 Critical Assessment of Small Molecule Identification (CASMI) contest, participants were provided 208 challenges with fragmentation information and candidate query sets retrieved from ChemSpider [16]. Using fragmentation information alone, participants were able to rank between 24 (11.5%) and 70 (33.7%) of these 208 challenges correctly in first place [16]. However, combining this fragmentation information with other forms of information (e.g. references, retention time information) yielded up to 164 (78.8 %) challenges correctly ranked in first place when combining all participant methods over the same ChemSpider candidate sets [16]. Separately, a detailed evaluation of MetFrag combining retention time information with various scoring terms available via ChemSpider (5 different literature terms) and PubChem (PubMed Count and Patent Count) for 473 environmentally relevant standards was performed. This revealed that ranking results were improved from 22 to 89% with ChemSpider and from 6 to 71% with PubChem (with 34 and 71 million entries respectively at the time) [26]. In summary over these evaluations and more; better ranking performance is achieved with small, select databases, at the risk of missing the correct answer [28], while the use of additional metadata (expert knowledge, additional context) is necessary to improve the results for practical use, especially when using very large compound databases to search for candidates.

Another challenge, especially for exposomics, is database choice. Being a mix between metabolomics and environmental concepts and challenges, exposomics methods need, on the one hand, the biological context of pathway and metabolomics resources (generally small, specialist metabolite databases such as HMDB and KEGG), versus the wide coverage required to capture “chemical space” which, in environmental contexts, generally means PubChem or ChemSpider. Although recent works mention the need for an “exposomics database”, much of the necessary knowledge is already in the public domain to some extent, but under rapid development and scattered over an ever-growing number of resources. Notable recent developments include the CompTox Chemicals Dashboard, covering 882,000 (August 2020) environmentally and toxicologically-relevant compounds [21] and the Blood Exposome Database [29], which, although specifically designed for the blood matrix, still contains over 64,000 compounds. Large compound databases such as PubChem have content in common with many of the openly available smaller databases, but at a size of 109 million compounds (January 2021), PubChem also contains many (tens of) millions of entries that are not relevant to the exposomics context.

Beyond the database choice, common criticisms of small molecule identification coupled to compound databases arising from users over the years include the fact that newly-discovered and/or relevant compounds such as emerging chemicals, transformation products and metabolites are missing from, or hard to add to, these databases for a typical researcher. If these compounds are present, these tend to have very low metadata scores and thus common environmental knowledge of transformations or emerging chemicals cannot often be found effectively during identification efforts. As a result (and also to increase efficiency), many groups in the environmental community have taken to compiling their own lists of relevant chemicals (commonly termed “suspect lists” within this community [7]). The NORMAN Suspect List Exchange (NORMAN-SLE) [30] is one initiative that arose to address NORMAN Network [31, 32] member needs to exchange this information as a result of a collaborative trial in 2014 [33], and to date is host to over 73 specialised NORMAN member contributed lists of chemicals of interest.

With a view on this “current state”, this article investigates how very large compound databases, or knowledge bases, such as PubChem, could be empowered to support HR-MS-based small molecule identification efforts in the context of exposomics. This article describes initial collaborative efforts on how to improve the performance of the PubChem integration into the in silico identification approach MetFrag. Since the first release of MetFrag in 2010, PubChem has grown from 25 million to now 109 million compounds, with an accompanying steadily worsening rank performance and increasing strain on resources due to the rapidly increasing candidate numbers. Three main aspects of these collaborative discussions are presented in this article: (1) the creation of a small, exposomics-relevant subset of PubChem–named PubChemLite–for efficient candidate queries, which has already been integrated into existing HR-MS workflows and teaching efforts; (2) progressive integration of environmentally-relevant expert knowledge to mitigate identified knowledge gaps in PubChem annotation content, based on analysis of previous benchmarking sets and the NORMAN-SLE content; and (3) how annotation content can be leveraged for easier interpretation of results. As a result, this article focuses heavily on PubChem, MetFrag and the NORMAN-SLE, with the view that the ideas presented here could be extended to other knowledge bases and other in silico identification approaches based on HR-MS.

## Results and discussion

### Creating “PubChemLite” for exposomics

**Fig. 1:**
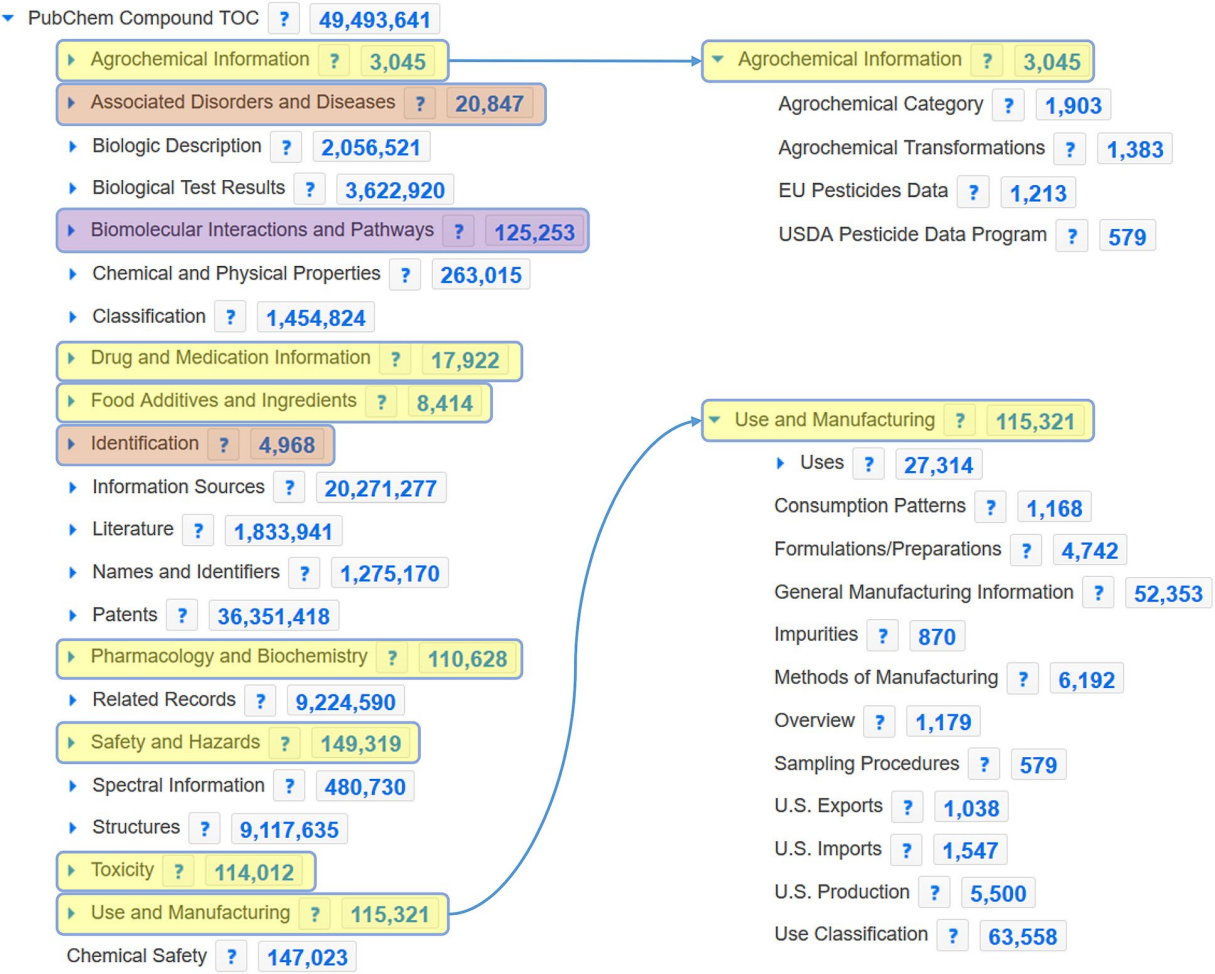
PubChem Compound Table of Contents (TOC) Tree (2 Nov. 2020) from the PubChem Classification Browser [34]. The contents (and categories) are updated regularly. Left: the top 22 categories (of the current total 524) are shown (default view). Yellow shading indicates the seven categories used in PubChemLite tier0 (“environmental” selection), the purple shading indicates the additional category used for PubChemLite tier1 (“exposomics”); red shading indicates the two categories that were added into the final PubChemLite exposomics selection. Right: Expansion of the “Agrochemical Information” and “Use and Manufacturing” sections as examples of sub-categories

Since a very large proportion of the PubChem database (> 60%) is sourced from purchasable screening libraries from chemical vendors, where the chemicals are generally produced in relatively small amounts (e.g. mg) in a laboratory setting, the vast majority of these chemicals are highly unlikely to be detectable in either the environment or biological samples. Thus, instead of the current status quo, i.e. searching the entire PubChem database and using metadata scores to “up-prioritize” interesting candidates (i.e., processing tens of thousands of candidates per mass, to only obtain tens to hundreds of interesting entries), the first step investigated the creation of relevant subsets of PubChem for more efficient queries. This was done by selecting relevant sections of the “PubChem Compound Table of Contents” (PubChem Compound TOC) Classification [34] as shown in Fig. 1. Further details are given in the "Methods" section.

**Fig. 2:**
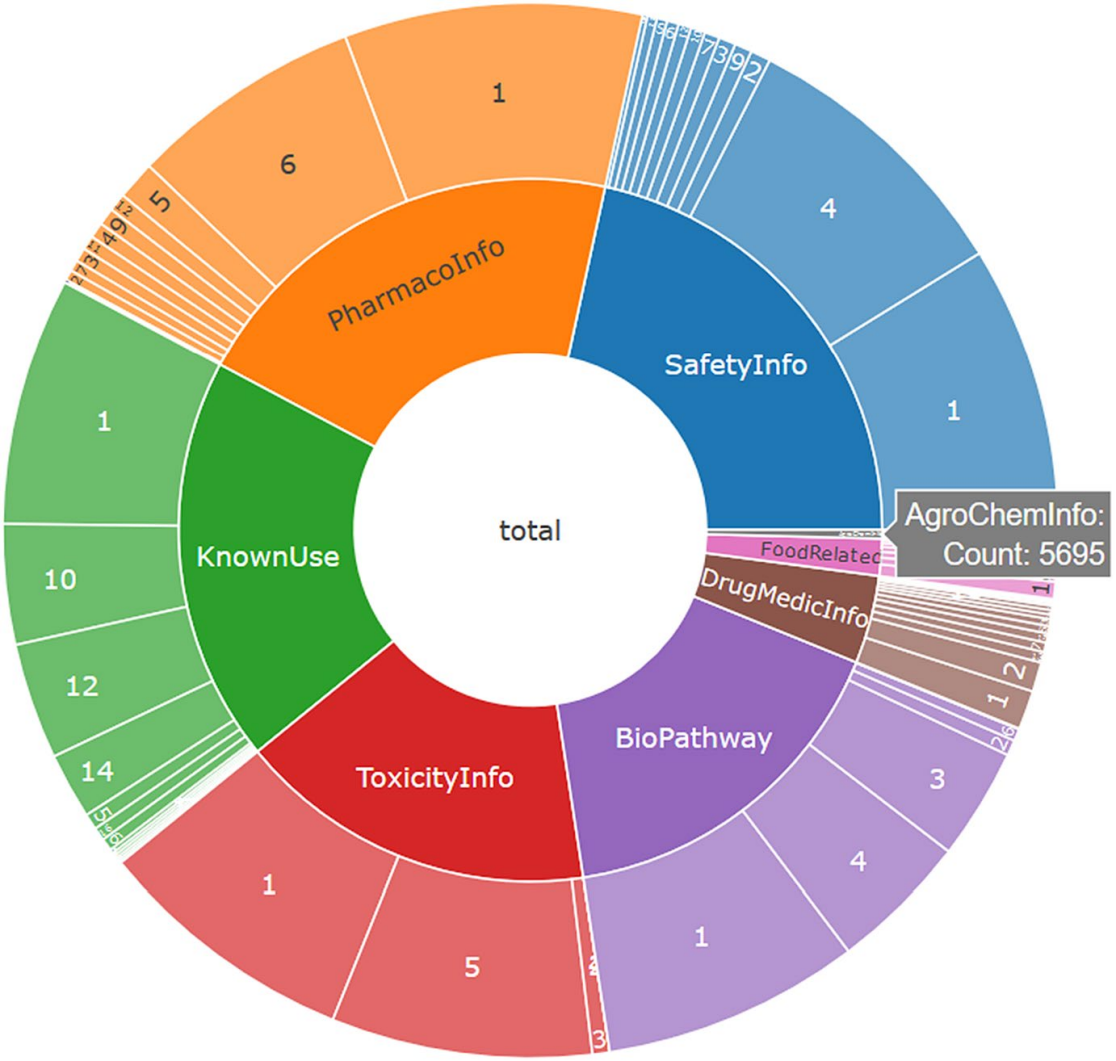
Sunburst plot of PubChemLite (14 January 2020 tier1 version [36]) to visualise the content. Note many CIDs are in multiple sub-categories, and total counts include this duplication (i.e. the 5695 AgroChemInfo count corresponds with fewer unique CIDs, see below). An interactive version embedded in an RMarkdown file is available as Additional file 1, the interactive plot plus code and example file is also available on the ECI GitLab pages [37, 38]

Initially, two versions of PubChemLite were created. The environmental selection (PubChemLite tier0), formed of the yellow-shaded categories in Fig. 1, shortened to “AgroChemInfo, DrugMedicInfo, FoodRelated, PharmacoInfo, SafetyInfo, ToxicityInfo, KnownUse”, whereas the exposomics selection (PubChemLite tier1) had the additional purple-shaded category, shortened to “BioPathway”, which contained the additional biological information categories relevant to metabolomics and exposomics. Entries were merged by InChIKey first block (the structural skeleton), and total Patent Counts and Literature Counts were calculated over the merged entries (full details in the "Methods" section). Each category was added as an additional column, where each entry was assigned a value that was a (merged) count of the sub-categories, and a total annotation count column was also added, summing the presence in top categories only (for further details, see "Methods"). Initial versions (20 November 2019 [35]/14 January 2020 [36]) contained 315,843/316,810 entries in tier0 (environmental collection) and 361,976/363,911 entries in tier1 (exposomics). In other words, the 103 M entries of PubChem (at the time) were collapsed down to two datasets of approximately 316 K and 360 K compounds. An RMarkdown file to visualize the content (categories and subcategories) of PubChemLite as an interactive sunburst plot (for a static version see Fig. 2) using the 14 January 2020 tier1 version is included as Additional file 1 and is also available on the ECI GitLab pages [37, 38]; further details are in the "Methods" section below.

**Fig. 3:**
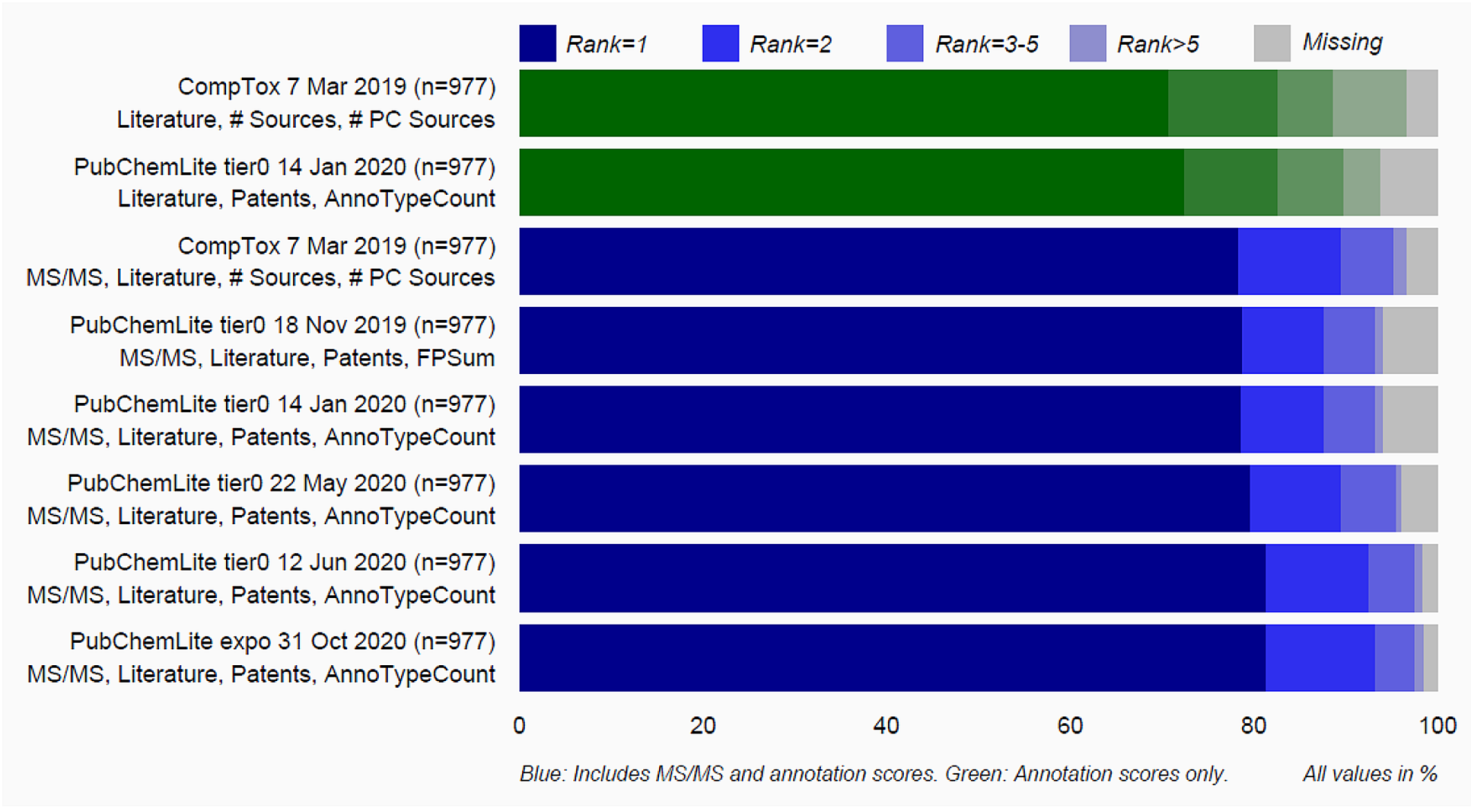
The ranking performance of various versions of PubChemLite versus CompTox using the merged benchmarking set (n = 977) with comparable metadata terms. Green: without MS/MS information. Blue: with MS/MS information (includes in silico fragmentation and MoNA library scoring terms). The increase in top ranks and decrease in missing entries with newer versions shows the influence of additional annotation content in PubChem (see Section “Identifying and Filling Gaps in PubChem Annotation Content”). The script and associated data files to reproduce this plot are available on the ECI GitLab pages [41, 42]. Figure template from [43]

A benchmark dataset of 977 de-duplicated compounds (see Additional file 2) was created by merging chemicals from previous evaluations [16, 26] (predominantly environmentally relevant) as described in "Methods". MetFrag was run with different versions of PubChemLite as well as CompTox (7 March 2019 release [39]) using comparable scoring terms. A summary of the results shown in Fig. 3 includes calculations both without (green) and with (blue) the use of MS/MS information (in silico fragmentation score and MS library matching scores). Further parameter details are given in the "Methods" section, with tables included in Additional file 3. Overall, CompTox and PubChemLite perform comparably; initially CompTox had fewer missing entries (grey shading) due to their earlier concerted efforts to add compounds of environmental interest, including transformation products (these gaps may well be smaller with the new data release). These gaps were closed progressively in PubChemLite as described in the next section “Identifying and Filling Gaps in PubChem Annotation Content”. Furthermore, early results (see Additional file 3: Figures S1 and S2, Tables S1 and S2) showed that both versions of PubChemLite, tier0 and tier1, performed almost identically even on environmental substances of interest, such that finally, one “PubChemLite” for exposomics was created, equivalent to tier1 plus the two additional categories as shown in Fig. 1 [40]. Results from this version are also shown in Fig. 3.

The results in Fig. 3 show that, while annotation information alone leads to good ranking performance (~ 70–73% ranked first, dark green shaded results), the MS/MS information is essential for further improvements (~ 79–83% ranked first, dark blue shaded results). This is discussed further below. The PubChemLite results on the two initial versions (20 November 2019 and 14 January 2020) also clearly show that ~ 8 % of the benchmark dataset were missing from PubChemLite. A detailed interrogation of the benchmark set of 977 reference standards from Eawag and UFZ revealed that—as commented by the community over many years—detailed annotation information was missing for well-known relevant transformation products in PubChem. This accounted for 37 of the 57 missing entries in the January 14, 2020 tier0 version and is discussed further in the next section.

### Identifying and Filling Gaps in PubChem Annotation Content

During previous evaluations of MetFrag specifically [26], and in silico identification approaches for HR-MS in general during e.g. CASMI [16], the focus has generally been on evaluating the methods themselves, aiming for objective evaluation. The use of identification approaches in typical real-life scenarios, however, often requires additional subjectiveness to provide *interpretation*, not just *identification*. Thus, the material in this article should not be viewed as an evaluation of MetFrag itself (which has not changed), but rather demonstrates how improving the underlying database and associated functionality can help to improve outcomes for users (i.e. the ability to find relevant chemicals) in the context of exposomics. In other words, this has been an opportunity to investigate and improve the annotation content (i.e. information content beyond structural properties) in PubChem for exposomics.

**Fig. 4:**
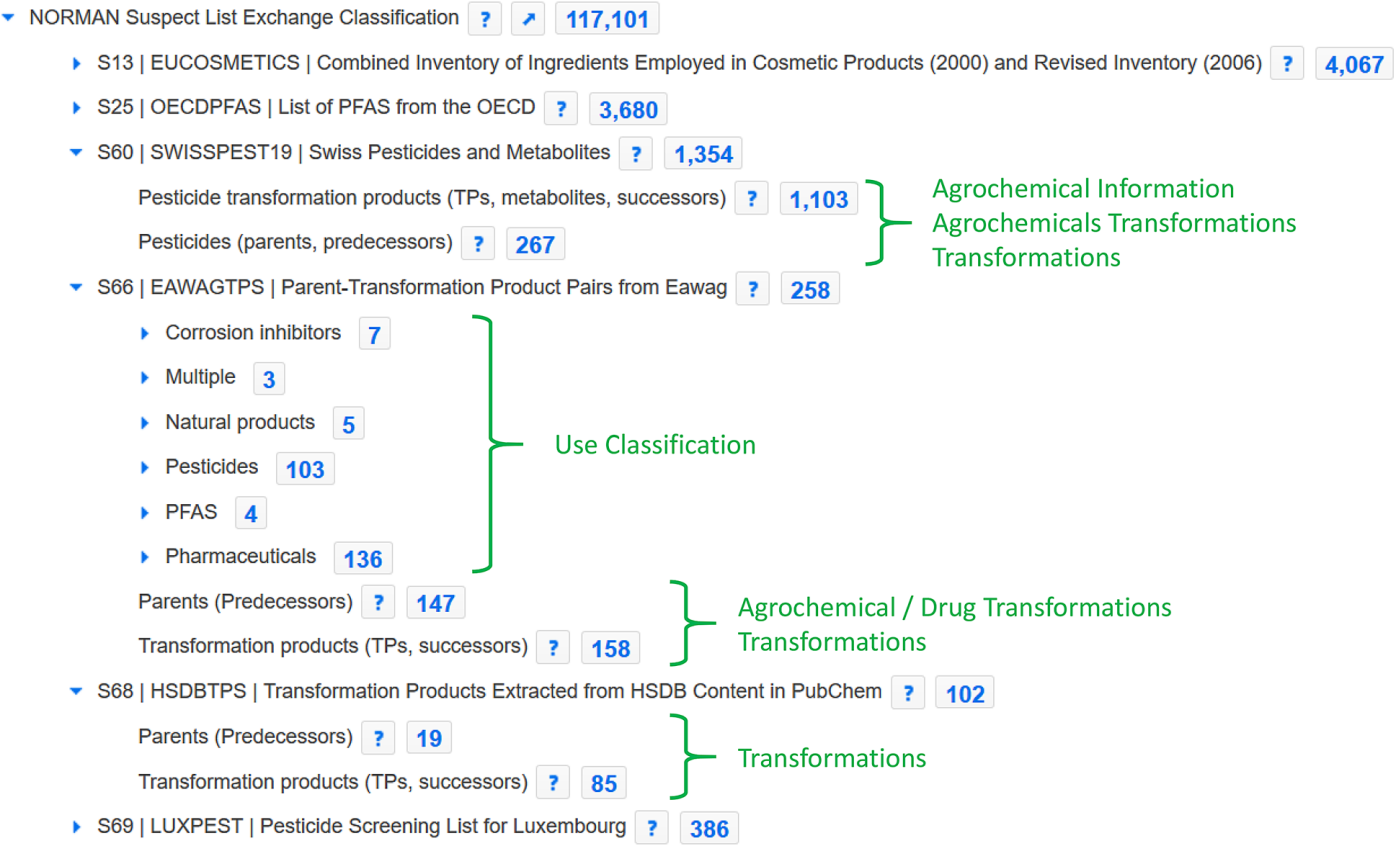
Screenshot of the NORMAN Suspect List Exchange Classification in PubChem (13 August 2020), including (partial) expansions of the S60, S66 and S68 lists, with the corresponding sections added to individual records indicated in green type

**Fig. 5:**
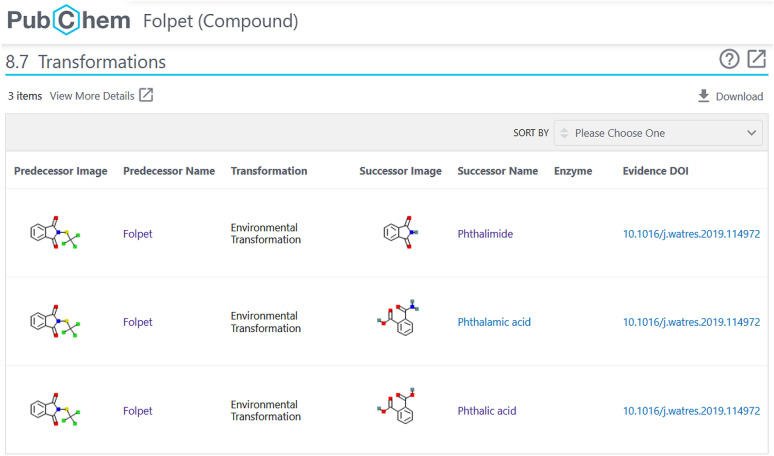
Transformations section in PubChem for Folpet (CID 8607) from SWISSPEST19 [46]

As Fig. 3 reveals, 57 chemicals from the benchmark set were missing in the early versions of PubChemLite, many of which were well-known transformation products in environmental studies. Since adding annotation content requires also sufficient provenance and evidence to support the annotation, the NORMAN-SLE [30, 44], which now has its own Classification Browser [45] in PubChem (see Fig. 4) was browsed for suitable suspect lists containing annotation content. Initial efforts concentrated on list S60 (SWISSPEST19) [46], a list of pesticides and transformation products/metabolites documented by Kiefer et al. [47]. This list contained parent-transformation product mappings, plus the link to information about agrochemical use (since the focus was on pesticides). The list was modified into a “predecessor/successor” mapping form (to avoid terminology clashes within other sections of PubChem) and added, with full provenance, into a new “Transformations” section in the individual PubChem records (see Fig. 5). Accompanying statements on “Agrochemical Transformations” within the agrochemical sections were also added, for example “Folpet has known environmental transformation products that include Phthalimide, Phthalamic acid, and Phthalic acid” [48]. The PubChemLite version created 22 May 2020 [49] included these new annotations, with fewer missing entries and slightly better ranks (see Fig. 3). Since this only focused on the agrochemicals (pesticides), the many pharmaceutical (and other) transformation products among the Eawag dataset were still missing. While these are all present in MassBank [50] (S1 in the NORMAN-SLE [51]), this dataset does not come with appropriate annotation content or provenance. Instead, the Supporting Information from Schollee et al. [52] provided suitable parent-TP mappings to create the predecessor-successor tables, which was merged with the Eawag classification information (with permission and support from Juliane Hollender) and added as list S66 [53]. This collection, together with list S68 HSDBTPS [54], resulted in the greater coverage in the June 2020 [49] and October 2020 [40] versions (see Fig. 3), with only 16 missing entries (15 in October) remaining. These remaining 16 entries could not be clearly related to any specific NORMAN-SLE lists to add further annotation content at this stage; although annotation content is being progressively added in separate efforts—as is evident from the one less missing entry in October.

### Leveraging annotation content in exposomics

**Fig. 6:**
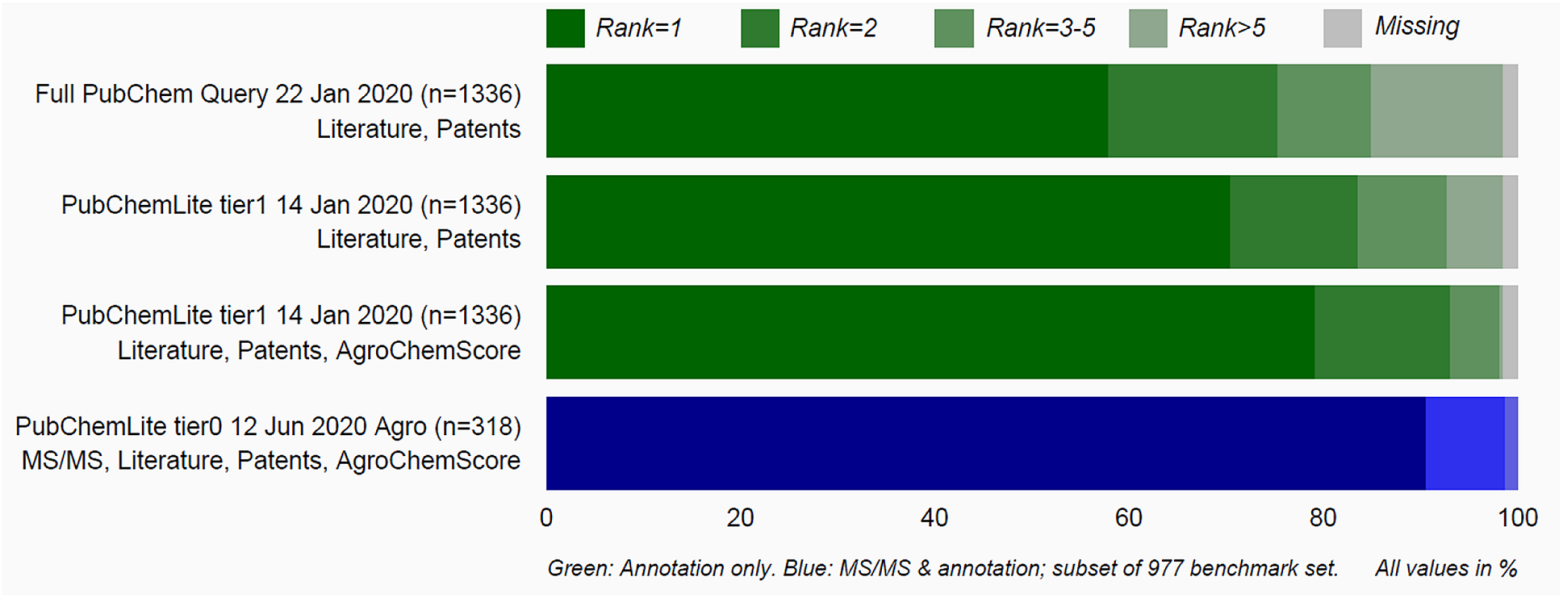
Green shading: Ranking performance of PubChemLite (14 Jan. 2020) in MetFrag using annotation score alone (no MS/MS information) with the Agrochemicals set from 14 Jan. 2020. Top: full PubChem (live query, 22 Jan. 2020 with 102,404,298 compounds). Second: PubChemLite tier1 with literature and patent scores and third: with the addition of the AgroChemScore (number of subcategories of agrochemical information available). The AgroChemScore is not (yet) available for the full database. Note: missing agrochemical entries are due to the presence of metals in some agrochemicals, which are excluded from MetFrag results (see "Methods" for rules applied to create PubChemLite). Bottom in blue shading: Ranking performance of PubChemLite (12 Jun. 2020) in MetFrag using topic-specific annotation score plus MS/MS information on the subsets of the benchmarking containing agrochemical annotation information. The script and associated data files to reproduce this plot are available on the ECI GitLab pages [41, 42]. Figure template from [43]

The results presented in Fig. 3 detailed the use of rather generic metadata terms (literature counts, patent counts, total annotation counts). However, one aim of setting up PubChemLite was not only to merge several “useful” categories for exposomics, but to leverage the information within these categories (providing *interpretation* about candidates in candidates sets). The smallest annotation category in PubChemLite, the agrochemicals, was taken as an additional benchmarking dataset (1336 chemicals, 22 Jan. 2020, see Additional file 4) to investigate the influence of database size and the additional scoring terms on the ranking results. Since this was to mimic an environmental investigation interested in detecting agrochemicals (i.e. a “suspect screening” approach [7]), the “agrochemical score”, i.e. how many agrochemical categories exist in PubChem for that chemical, was used as an additional scoring term in MetFrag (details in "Methods"). The results are shown as the green entries in Fig. 6; the exact numbers are given in Additional file 3: Table S3.

With a full PubChem query and using only literature and patent information to score, only 58% of entries were correctly ranked in first place (which is not unexpected, as e.g. pharmaceuticals, industrial chemicals or even metabolites with the same mass may have larger literature or patent counts). When the database was restricted to the candidates in PubChemLite using the same scoring terms (literature and patent counts), this increased to 70%. However, adding the Agrochemical Score improved this further to 79.2 %, demonstrating the potential usefulness of individual category-based scoring terms to help select relevant chemicals for further verification. In terms of computational efficiency, the last 101 queries (entries 1236–1336) of the Agrochemicals query took 11 min to complete with PubChemLite tier1 (query run 21 Jan. 2020), while the equivalent query with the full PubChem database and scoring terms took 164 min (query run 26 Jan. 2020). This results in approx. 6.5 s per query for PubChemLite, versus 97 sec per query for a full PubChem query (note: both queries were without fragmentation).

Since this is purely annotation-based scoring, it is imperative to use additional experimental information such as fragmentation information and further verification with reference standards before any claims of higher confidence annotation are made [11]. To address this, the benchmarking dataset (n = 977) used above (with MS/MS information available) was subset according to the availability of information in the Agrochemical Information category (creating a subset of n = 318), and evaluated with scoring terms relevant to the annotation type, as shown in the blue entry in Fig. 6. This mimics, to a certain extent, a typical suspect screening workflow where the main interest is in finding and confirming pesticides in an environmental sample. As shown, adding MS/MS information (MetFrag in silico fragmentation plus MoNA similarity score) increased the correctly ranked chemicals in first place to 90.6% for those agrochemicals that were also in the benchmarking set. If the database (in this case PubChemLite tier0 12 Jun. 2020 version) had been restricted to agrochemicals only this would have risen to 94.3%, as some non-agrochemical isomers still outscored several entries based on the literature and patent values. The performance would not be able to rise much higher than 94% with this dataset, however, since there are multiple agrochemical isomers present in the dataset where the less-well-known (but often structurally related) isomers ranked lower because of less supporting metadata. For instance, for secbutylazine (CID 23712), the candidate terbutylazine, CID 22206 was ranked first and secbutylazine, CID 23712 was third, while another isomer propazine CID 4937 was second. All three isomers were in the dataset. In this case, both the in silico fragmenter and MoNA similarity scores captured these three isomers in the correct order (secbutylazine first, terbutylazine second, propazine third), showing that the experimental evidence is still crucial in distinguishing isomers - or indicating whether they are indistinguishable on given evidence. Terbutylazine was correctly ranked first for its corresponding entry (see Table 1).

**Table 1:** Candidate score distributions for three isomers/isobars of formula C_9_H_16_ClN_5_ in the agrochemical dataset

Name (CID)	Terbutylazine (22,206)	Propazine (4937)	Secbutylazine (23,712)
MetFrag Scores	*4.96*; 3.45; 2.77; 1.93; 1.59	4.46; *3.88*; 2.27; 1.81; 1.58	4.96; 3.52; *2.78*; 1.92; 1.57
Fragmenter Score	*351*; 250; 351; 239; 126	247; *295*; 251; 170; 106	398; 303; *403*; 272; 135
MoNA Similarity	*0.959*; 0.672; 0.987; 0.0; 0.0	0.638; *0.841*; 0.661; 0.0; 0.0	0.971; 0.703; *0.998*; 0.0; 0.0
PubMed Count	*282*; 127; 0; 11; 1	282; *127*; 0; 11; 1	282; 127; *0*; 11; 1
Patent Count	*10,935*; 8900; 1990; 6636; 6861	10,935; *8900*; 1990; 6636; 6861	10,935; 8900; *1990*; 6636; 6861
Annotation Count	*5*; 5; 4; 4; 5	5; *5*; 4; 4; 5	5; 5; *4*; 4; 5
AgroChemInfo	*5*; 4; 3; 3; 3	5; *4*; 3; 3; 3	5; 4; *3*; 3; 3
Rank	1 of 37	2 of 37	3 of 37

Values for the correct candidate in each case are italiczed. Only the scores for the top 5 candidates (of 37) are shown

Using this benchmarking dataset alone, taking PubChemLite and using the specific topic information for agrochemicals, most candidates were ranked 1st and the worst rank for a chemical was 3rd. Creating a similar pharmaceutical subset (as opposed to agrochemicals) using the “DrugMedicInfo” category yielded similar results (most ranked first, worst rank of 3rd ) using either DrugMedicInfo or PharmacoInfo as scoring terms (see Additional file 3: Figure S3). For a more generic category such as ToxicityInfo, most were ranked 1st or 2nd, but the worst rank was 12, indicating that this term may be less selective (see Additional file 3: Figure S3). Using patent and literature information alone (over the entire benchmark set), the worst rank was 27th, with 11 entries missing entirely. Thus, even though this dataset is of limited size (977 entries), the results indicate that there is a good chance that the top candidate will be among the Top 3 using PubChemLite for highly specific categories such as (agrochemicals, pharmaceuticals). On the other hand, more candidates will often have to be considered for less specific categories or questions (e.g. Toxicity Information) or when only the generic scoring terms are used. In the context of practical use of HR-MS for answering real life questions, e.g. the presence of well-known chemicals in environmental or patient samples, considering only a few candidates (e.g. 1–3) versus hundreds or even thousands of candidates per mass is a great step forward for higher throughput *interpretation* of non-target screening results and coming to meaningful conclusions quicker. It is expected that greater granularity in the annotation information will improve the interpretability and applicability of this information in the future (for instance toxicity information is currently often only “information is present” and not “the substance is toxic”); efforts are being made to achieve this (beyond the scope of the current article). Regular updates/deposition of relevant third-party data resources in PubChem such as HMDB, CompTox and the Blood Exposome database will help ensure that this content can be included and updated in PubChemLite.

As a future perspective, the addition of extra information, such as partitioning information (e.g. log*P*, log*K*_*ow*_ or log*D*) and collision cross section (CCS) values, will also help in candidate selection in specific cases (although for isobars /isomers that are very similar, predictive values will often be very close). Efforts are currently underway to include XlogP3 [55] in future versions of PubChemLite to integrate within the retention time model already present in MetFrag [26]. Further, an initial version of PubChemLite (January 14, 2020 tier1) with CCS values contributed by CCSbase [56, 57] is also available on Zenodo [58] and in MetFrag web version [27] and is currently being evaluated in separate work.

## Conclusions

The need to cover the “entire chemical space” in exposomics research is a huge challenge for researchers and database resources alike (and currently unachievable – due to our inability to define chemical space completely). This article explores the use of annotation content of very large compound databases, i.e. compound knowledge bases, to create meaningful and efficient subsets relevant to specific use cases, specifically aimed at creating subsets of PubChem most relevant for exposomics. The resulting PubChemLite is a dynamic yet efficient database that grows as the respective (and relevant) annotation categories grow in PubChem, and is built and deposited regularly to allow integration with existing HR-MS identification approaches such as MetFrag [27, 59] and comprehensive MS workflows such as patRoon [60]. The subcategories present in PubChemLite allow end users a certain a degree of individual or sample-wide interpretation of the results, such that broad chemical categories become obvious amongst suggested candidates. These can be used as scoring terms or hard filters, depending on user choice, and subsets of the database could serve as large suspect lists if desired. PubChemLite is already in use in several research projects. Feedback on the approach and further integration into other resources and workflows is greatly welcomed. Further developments are being made behind the scenes to streamline the ideas presented in this manuscript for the community in other ways. The code and all necessary files are available (see availability statement), such that expert users can build and compile their own subsets of PubChem using any of the categories available in the PubChem Table of Contents Classification Browser [34] by defining their own input “bit sets”.

To address the “data gap” issue of highly-relevant compounds missing in existing compound databases (a broadly acknowledged weakness and argument frequently applied against using compound databases for HR-MS-based tentative identification efforts), this article also explores how knowledge gaps can be assessed and filled, as exemplified with environmentally-relevant information from the NORMAN Network. A coupled deposition and annotation workflow has been set-up between PubChem and the NORMAN-SLE, allowing the deposition of environmentally relevant substances into PubChem and the progressive integration of the accompanying (relevant) annotation content, with full traceability to the original data sources. The examples covered in detail here included transformation product and agrochemical use cases. Importantly, these integration efforts enhance both resources and help combine knowledge into a central location (thus increasing the FAIRness of the data) by reducing the isolation of the individual NORMAN-SLE lists while increasing the annotation (information) available in PubChem. The integration of content is occurring progressively with a focus on areas of high community interest and on those filling the largest gaps. Community input is very welcome to help focus these efforts to maximize the overall benefit. The content is available in a variety of formats across both resources for re-use.

While PubChemLite is an immediately accessible stepping-stone for HR-MS-based exposomics research, it is still only a small part of efforts towards a bigger picture solution for the exposomics challenge. Enhancing the annotation content of compound knowledge bases is clearly one way of improving the useability of very large knowledge bases. Dynamic and easy-to-use ways to subset and/or order the chemicals based on this annotation content (beyond creation of a MetFrag-specific output file) will be needed to improve the useability further. At some point, specialist users will need to be able to tell chemical knowledge bases what they want to find to improve their search results for their specific use case, rather than just taking the “best match” based on generic scores such as literature or annotation counts. Future efforts, beyond enhancing annotation content, will include continuing conversations with users and the community to develop functionality that can be applied either on the database side, or the workflow side, or both, to truly empower large compound knowledge bases for exposomics research and move from just *identification* towards more detailed *interpretation* of HR-MS datasets.

## Methods

### Creating PubChemLite for MetFrag

MetFrag currently has PubChem integrated via the RESTful API as well as a local mirror. Of the typically thousands of candidates that are retrieved using exact mass (with ppm error margin) or molecular formula queries, several candidates are returned that are eventually discarded (e.g. disconnected structures, which cannot be observed at the input mass or formula in the mass spectrometer, or other structures that cannot be processed by MetFrag). Since high resolution mass spectrometry rarely yields information on stereochemistry (there are exceptions for some substances e.g. when chiral chromatography is used), it is the default behaviour of MetFrag and many other approaches to merge candidates by the first block of the InChIKey (i.e. the structural skeleton) and present the users results displaying the stereoisomer with the highest score. For candidates merged by InChIKey first blocks, any ranking is usually driven by metadata rather than fragmentation, which does not usually contain sufficient information to distinguish stereoisomers, except for some tautomers. In MetFrag, this stereoisomer filtering can be switched on or off as desired. However, for larger (or complex) structures, the presence of stereoisomers can dramatically inflate candidate numbers and reduce calculation efficiency, often for little final gain.

To create subsets of PubChem by annotation content category, firstly a Table of Contents fingerprint (TOC FP) was created for each of the PubChem Compound TOC entries (each bit representing presence or absence of information in that category for a compound) along with metadata indicating the relationship between the bits (e.g., subcategories of a given annotation). Then, mapping files containing the desired TOC entries were created. Finally the relevant data (compound information, patent and literature scores, plus the TOC fingerprints) was extracted by the compound identifier (CID) from the respective PubChem download files [61] using scripts that have been made available at the Environmental Cheminformatics group GitLab pages [62].

Following this, and considering the current, established MetFrag behaviour [26], a set of rules was applied to the CIDs extracted from the TOC categories to generate a file that could be processed by MetFrag. Candidates that would be discarded later anyway (e.g. disconnected structures or other structures that cannot currently be processed by MetFrag) were discarded up front. Further, CIDs were collapsed by the first block to have one “best matching” CID and mappings to all related CIDs. The rules applied were the following:

1. Retrieve all CIDs in PubChem with the desired annotation categories;
2. Map all CIDs to corresponding parent CIDs to obtain the neutral form, where available, imputing the annotation to the parent;
3. Collapse by InChIKey first block (IKFB), imputing total annotation to the IKFB, retaining the “best” CID (the most annotated CID for the given IKFB) and listing all related CIDs in a separate column, thus grouping all CIDs with annotation available;
4. Remove all entries containing the following elements: Kr, Dy, Ir, La, Lu, Nd, Nb, Os, Pd, Pt, Pu, Pr, Re, Rh, Ru, Sm, Sc, Ag, Ta, Tc, Tb, Th, Tm, Ti, W, Ac, Am, Er, Eu, Gd, Hf, Ho, Xe, Yb, Rn, Sr, Be, Cm, Cf, Cs, Md, Pm, Fr, Pa, Np, Bk, Es, Fm, No, Lr, Rf, Db, Sg, Bh, Hs, Mt, Ds, Rg, Cn, Nh, Fl, Mc, Lv, Ts, Og;
5. Remove disconnected structures-as these will not be observed at the mass/formula of the query;
6. Remove charges from charged molecular formulae (but not the corresponding structures).

These rules were selected for maximum efficiency, resulting in the following behaviour that should be considered when interpreting the results. Firstly, collapsing all annotated CIDs by IKFB could result in the inclusion of different isotopic states and/or charges, which may not be included otherwise in MetFrag queries initiated by exact mass/formula and could otherwise prevent these candidates appearing in PubChemLite queries at their true exact mass/formula. In the context of efficient screening of masses for environmental, metabolomics or exposomics studies, matches with differing isotopic states are unlikely to be found in large amounts in these studies. In the cases that isotopically labelled standards are used, or isotopically labelled experiments are performed, other data interrogation techniques are usually necessary/recommended to capture these peaks in advance of identification efforts. For differing charge states, since these are usually accounted for in the upstream workflow by adjusting the adduct state, the current behaviour ensures a consistent “base state” for adjustment of charge in other parts of the workflow. Secondly, mixtures are currently discarded from PubChemLite files, as this would require an additional degree of manipulation (splitting and re-merging of the entries), which was not accounted for in the current version as this affects < 10K entries - of which a significant proportion are salts. It would be possible to address both issues in future versions should subsequent use cases deem this necessary. Finally, related CIDs are only included if that CID contains any annotation in at least one of the selected annotation categories. For example, the InChIKey first block HXKKHQJGJAFBHI has 6 related CIDs in PubChemLite tier 0 (14 Jan 2020 version: 4, 111033, 439938, 446260, 7311736, 44150279), while 9 CIDs (4, 439938, 446260, 4631415, 7311735, 7311736, 16655457, 123598986, 140936702) match this InChIKey first block in the PubChem search interface (search date 22 May 2020 [63]).

As PubChem is changing daily, both in terms of numbers of chemicals and their annotation content, PubChemLite will not remain static. Initial evaluations in this paper were done on the first archived versions, generated November 18th, 2019 [35], with 640 category fingerprints generated on October 2nd, 2019. There were approximately 33 M entries with TOC annotations at this stage (e.g. 33,766,782 on October 29th, 2019). A second archived version, with additional scoring, was created January 14th, 2020 [36] for further evaluation. By this time the fingerprint consisted of 652 categories (January 9th, 2020) and there were 35 M entries with TOC annotations (35,800,159 on 21 January 2020). The third major version, PubChemLite for exposomics (31 October, 2020) was based on a fingerprint of 524 categories (29 October 2020) and there were 49 M TOC annotations (49,493,641 on 2 November 2020). A breakdown of these files is given in Table 2. These datasets are archived as versions 0.1.0, 0.2.0 and 0.3.0 on Zenodo [35, 36, 40].

**Table 2:** The breakdown of the major PubChemLite versions by InChIKey First Blocks (IKFB) and CIDs

	18 Nov 2019		14 Jan 2020		31 Oct 2020
	Tier0	Tier1	Tier0	Tier1	Exposomics
PubChemLite (by IKFB)	315,842	361,556	316,810	363,911	371,663
Eliminated (by IKFB)	12,056	12,762	11,979	12,682	12,971
Total (by IKFB)	327,898	374,318	328,789	376,593	384,634
Parent CIDs (or CID, if no parent)	377,278	430,246	378,581	432,645	431,067
CIDs with desired annotation	402,746	458,621	405,285	462,356	462,838

For the November 18, 2019 versions, an “FPSum” was calculated for all entries by adding the FP bits to give a maximum of 7 (tier0) or 8 (tier1). Individual columns for each annotation category were also created, so that the annotation categories could be used via the scoring term function in MetFrag, in addition to the patent and literature information. The resulting datasets (with preview) are available on Zenodo [35]. For the January 14, 2020 and subsequent versions, “FPSum” was modified to “AnnoTotalCount”, so the column name better reflected the content, i.e. the availability of annotation categories for that entry. Additionally, individual columns were created for each annotation category, filled with values calculated by adding the category plus the number of subcategories present for that annotation, which ranged from 3 to 15 subcategories (Jan. 2020). The resulting datasets are on Zenodo [36] and were integrated into the dropdown menu of local databases for MetFragWeb [27]. PubChemLite was built approx. weekly following the January 14, 2020 format to test systems, with two versions used in this article to check additional annotation content (see results and [49]). During evaluations, it became clear that two additional categories would be useful, one being “Identification” (present but previously overlooked) and the second being “Associated Disorders and Diseases” (not present when PubChemLite was officially drafted). Based on the evaluations showing little difference between tier0 and tier1, one version equivalent to tier1 plus these two additional categories has been built and released as “PubChemLite for exposomics” version 0.3.0 [40] and integrated into MetFragWeb [27] and patRoon [60, 64]. Subsequent updates will be built and auto-committed to Zenodo (after passing build checks) to allow automatic updates for MetFragWeb [27] and any workflows/users of the MetFrag command line (MetFragCL) version [59] and other workflows like patRoon [60].

### Assessing PubChemLite

The performance of PubChemLite was assessed using various datasets that were already used to evaluate MetFrag performance; CASMI 2016 [16] and MetFrag Relaunched [26] (hereafter MetFragRL). The CASMI2016 dataset consisted of 208 compound-MS/MS spectra pairs. The MetFragRL evaluation sets consisted of four groups of spectra measured under different conditions (datasets EA, EQEx, EQExPlus and UF, with n = 473, 289, 310 and 226, where n refers to the number of compound-MS/MS spectrum pairs). The calculations performed on the individual datasets are presented in Additional file 3: Table S1 and Figure S1, alongside the previously published results. Since some compounds had mass spectra available in both modes, and there was some overlap between the different datasets, this corresponded to a total of 1298 (MetFragRL) and 1506 (MetFragRL + CASMI) compound-MS/MS pairs overall. Calculations performed on this set (comparing PubChemLite tiers and CompTox) are presented in Additional file 3: Table S2 and Figure S2. For the purpose of clarity in the main manuscript, this set of 1506 was de-duplicated down to a set of 977 unique compounds by InChIKey First Block after accounting for multiple tautomeric forms, to eliminate any confusion due to the presence of duplicate spectra/modes. The MS/MS spectrum record number (the first-matching entry in the case of multiple spectra) was used to automatically extract and save the corresponding MS/MS peaks into the file using an R script, using the MS/MS spectra provided as SI for the respective studies, downloaded from the journal pages [16, 26]. As all compounds were present in PubChem, additional compound information was filled in using PubChem web services via R functions. The final benchmarking file (hereafter “PCLite Benchmark” set) is available as Additional file 2 and on the ECI GitLab pages, along with all associated code [62].

The PCLite Benchmark set was used to evaluate various versions of PubChemLite (dates: 18/11/2019 [35], 14/01/2020 [36], 22/05/2020 [49], 12/06/2020 [49] and 31/10/2020 [40]) as well as the CompTox Chemicals Dashboard version from 7/03/2019 archived as MetFrag Local CSV (database) files [39, 65]. Files are not yet available from the most recent CompTox release (but have been requested). The “Select Metadata” version of CompTox was used, which contained 857,615 entries, corresponding to 773,561 DTXCID InChIKeys and 773,232 InChIKey First Blocks associated with DTXCIDs (the CompTox “MS-ready” form [66] of information used in MetFrag). All CompTox files from the given release contain the same number of entries, just with varying metadata content. All queries were run with exact mass plus 5 ppm error, additional scoring terms and other parameters as detailed in Additional file 3: Table S4 and in the supporter scripts available on the ECI GitLab pages [67].

## Supplementary Information

**Additional file 1.**  Visualisation of PubChem Compound TOC Content in PubChemLite (RMarkdown).

**Additional file 2.** The merged benchmarking set of 977 compounds used in main text (CSV).

**Additional file 3.** Additional tables and figures to support the main document.

**Additional file 4.** The agrochemical benchmarking set (CSV).

## Data Availability

All the files needed to generate PubChemLite are available and updated at least weekly on the PubChem FTP website (https://ftp.ncbi.nlm.nih.gov/pubchem/) [61], all code to create PubChemLite with selected bit lists is available from the Environmental Cheminformatics group GitLab repository (https://git-r3lab.uni.lu/eci/pubchem/-/tree/master/pubchemlite) [62]. Fixed versions of PubChemLite mentioned in this manuscript are all archived on Zenodo [35, 36, 40, 49]. PubChemLite will be created and deposited to Zenodo at regular intervals following automatic checks [69], to allow integration with MetFrag [27], and offer download files for external users. The annotation content of the NORMAN-SLE (https://www.norman-network.com/nds/SLE/) [30] is being progressively added to PubChem [45], with all data available on PubChem [68] and Zenodo (https://zenodo.org/communities/norman-sle) [44]. The addition of new substances deposited to the NORMAN-SLE to PubChem is automated through mapping files and updated monthly (or more regularly if needed).

## Abbreviations

API: Application Programming Interface
CASMI: Critical Assessment of Small Molecule Identification
CCS: Collision Cross Section
CID: PubChem Compound Identifier
CompTox: US EPA CompTox Chemicals Dashboard
DAG: Directed Acyclic Graph
DTXCID: DSSTox Compound Identifier (from CompTox)
DTXSID: DSSTox Substance Identifier (from CompTox)
ECI: Environmental Cheminformatics group (at the University of Luxembourg)
FPSum: Addition of fingerprint bits to form a scoring term used in PubChemLite
HMDB: Human Metabolome Database
IKFB: InChIKey First Block
KEGG: Kyoto Encyclopedia of Genes and Genomes
MetFragRL: MetFrag Relaunched
MoNA: MassBank of North America
MS/MS: Tandem Mass Spectrum, MS2
NCBI: National Center for Biotechnology Information
NORMAN-SLE: NORMAN Suspect List Exchange (NORMAN-SLE)
PCL, PCLite: PubChemLite
RAM: Random Access Memory
SLE: Suspect List Exchange (see NORMAN-SLE)
TOC: PubChem Compound Table of Contents(PubChem Compound TOC)
TOC FP: Table of Contents fingerprint (TOC FP)
TOC Tree: PubChem Compound Table of Contents (TOC) Tree
