# Supplementary material for: Empowering large chemical knowledge bases for exposomics: PubChemLite meets MetFrag
Source: J Cheminform. 2021 Mar 8;13:19. doi: 10.1186/s13321-021-00489-0 (PMC7938590; doi:10.1186/s13321-021-00489-0)
Supplement: Supplementary file 3 — Additional file 3. Additional tables and figures to support the main document. [file 13321_2021_489_MOESM3_ESM.docx]

Additional File 3: Supporting Information for

Empowering Large Chemical Knowledge Bases for Exposomics: PubChemLite meets MetFrag

Emma L. Schymanski^1^*, Todor Kondic^1^, Steffen Neumann^2^, Paul Thiessen^3^, Jian (Jeff) Zhang^3^, Evan E. Bolton^3^*

^1^Luxembourg Centre for Systems Biomedicine (LCSB), University of Luxembourg, 6 avenue du Swing, 4367 Belvaux, Luxembourg. ELS*: [emma.schymanski@uni.lu](mailto:emma.schymanski@uni.lu) ORCID: 0000-0001-6868-8145. TK: [todor.kondic@uni.lu](mailto:todor.kondic@uni.lu) ORCID: [0000-0001-6662-4375](http://orcid.org/0000-0001-6662-4375)

^2^Leibniz Institute of Plant Biochemistry (IPB Halle), Bioinformatics and Scientific Data, 06120 Halle, Germany and German Centre for Integrative Biodiversity Research (iDiv), Halle-Jena-Leipzig Deutscher, Platz 5e, 04103 Leipzig, Germany. SN: [sneumann@ipb-halle.de](mailto:sneumann@ipb-halle.de) ORCID: 0000-0002-7899-7192

^3^National Center for Biotechnology Information, National Library of Medicine, National Institutes of Health, Bethesda, MD 20894, USA. EEB: [bolton@ncbi.nlm.nih.gov](mailto:bolton@ncbi.nlm.nih.gov)  ORCID: 0000-0002-5959-6190. PT: [thiessen@ncbi.nlm.nih.gov](mailto:thiessen@ncbi.nlm.nih.gov) ORCID: [0000-0002-1992-2086](http://orcid.org/0000-0002-1992-2086). JZ: [jiazhang@ncbi.nlm.nih.gov](mailto:jiazhang@ncbi.nlm.nih.gov) ORCID: [0000-0002-6192-4632](http://orcid.org/0000-0002-6192-4632)

Contents

[List of Tables and Figures 1](#_Toc55221703)

[Additional Results 2](#_Toc55221704)

[Benchmarking “PubChemLite” Against Previously Published Datasets and CompTox 2](#_Toc55221705)

[Leveraging Annotation Content 4](#_Toc55221706)

[Additional Methods 6](#_Toc55221707)

[List of Abbreviations 7](#_Toc55221708)

[References 7](#_Toc55221709)

# List of Tables and Figures

| Tables | | |
| --- | --- | --- |
| Table S1 | Benchmarking of PubChemLite against previously published datasets | 3 |
| Table S2 | Benchmarking of PubChemLite and CompTox | 4 |
| Table S3 | Performance of Annotation Scores with the agrochemical dataset | 4 |
| Table S4 | Summary of MetFrag parameters for all datasets (defined by supporter scripts) | 6 |
| Figures | | |
| Figure S1 | Benchmarking of PubChemLite against previously published datasets | 2 |
| Figure S2 | Benchmarking of PubChemLite and CompTox against previously published datasets | 2 |
| Figure S3 | Benchmarking of PubChemLite with Annotation Content | 5 |

# Additional Results

## Benchmarking “PubChemLite” Against Previously Published Datasets and CompTox


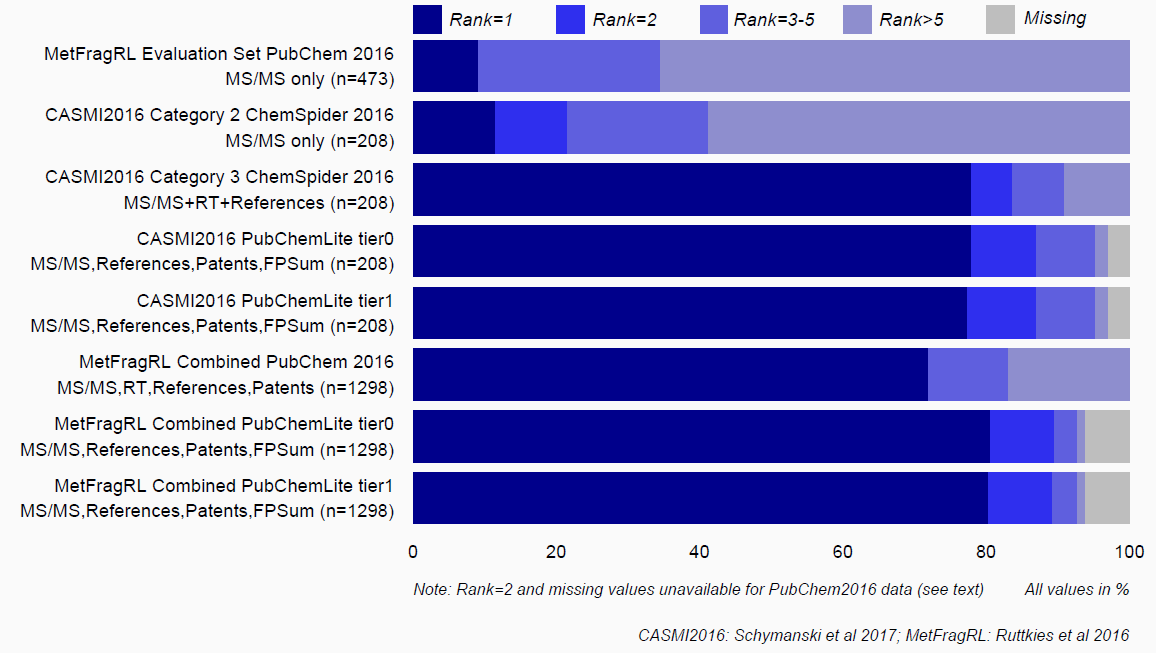


Figure S1: Benchmarking of PubChemLite (Nov. 18 2019 version [1]) against previously published datasets MetFragRL [2] and CASMI2016 [3]. Figure template modified from [4]. Script (plus link to data files) is available from [5].


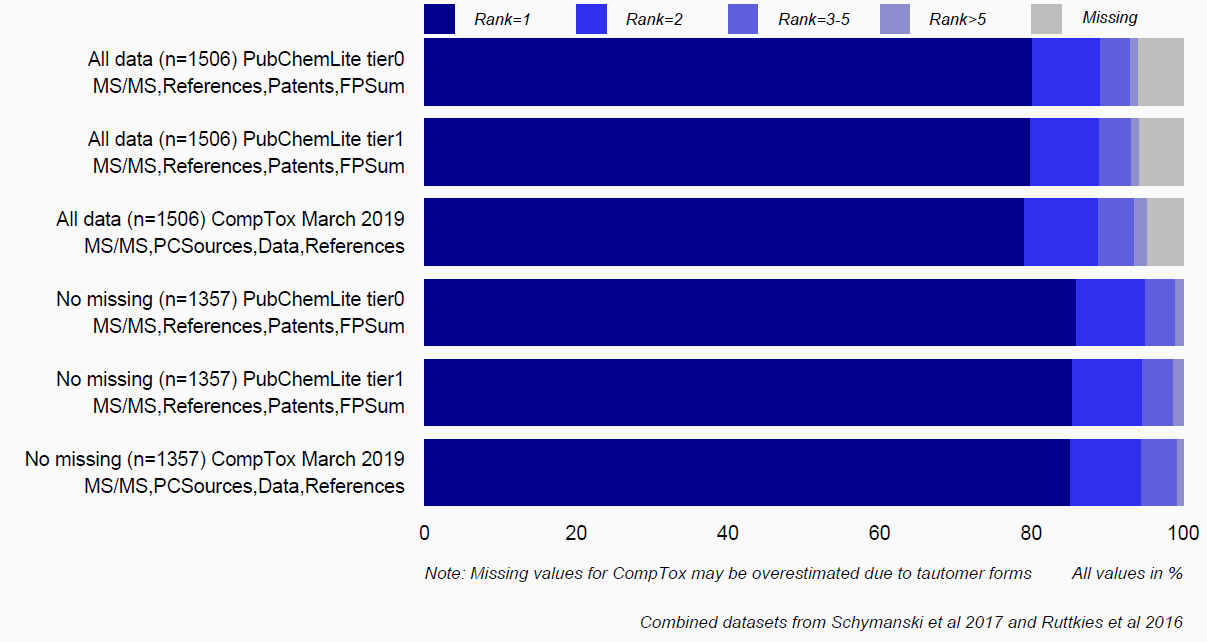


Figure S2: PubChemLite (Nov. 18 2019 version [1]) versus CompTox [6], with and without missing entries, using combined datasets from Figure S1 [2, 3], with the most comparable metadata terms and MS/MS information. Script (plus link to data files) is available from [5]. Figure template from [4].

Table S1: Benchmarking PubChemLite against previously published datasets. CASMI 2016 data and results were taken from [3]; MetFrag Relaunched data and results were taken from [2]. All PubChemLite calculations were performed on the November 2019 version [1] with parameters given in the methods.

|  | Top 1 (%) | Top 2 (%) | Top 5 (%) | Missing (%) |
| --- | --- | --- | --- | --- |
| ***CASMI 2016 Contest (n=208)*** | | | | |
| CASMI Category 2 MetFrag | 24 (11.5%) | 45 (21.6%) | 86 (41.3%) | 0 (0.0%) |
| CASMI Category 2 Combined | 82 (39.4%) | 105 (50.5%) | 144 (69.2%) | 0 (0.0%) |
| CASMI Category 3 MetFrag+RT+Ref | 162 (77.9%) | 174 (83.7%) | 189 (90.9%) | 0 (0.0%) |
| CASMI Category 3 Combined (MS/MS+RT+Refs+MoNA) | 164 (78.8%) | 181 (87.0%) | 190 (91.3%) | 0 (0.0%) |
| PubChemLite tier0 MS,Ref,Pat,FPSum | 162 (77.9%) | 181 (87.0%) | 198 (95.2%) | 6 (2.9%) |
| PubChemLite tier1 MS,Ref,Pat,FPSum | 161 (77.4%) | 181 (87.0%) | 198 (95.2%) | 6 (2.9%) |
| ***MetFrag Relaunched (Eawag EA n=473)*** | | | | |
| PubChem 2016 MSMS only | 43 (9.1%) | NA | 163 (34.5%) | NA |
| PubChem 2016 MSMS,RT,Ref,Pat | 336 (71.0%) | NA | 396 (83.7%) | NA |
| PubChemLite tier0 MS,Ref,Pat,FPSum | 357 (75.5%) | 401 (84.8%) | 424 (89.6%) | 38 (8.0%) |
| PubChemLite tier1 MS,Ref,Pat,FPSum | 357 (75.5%) | 402 (85.0%) | 425 (89.9%) | 36 (7.6%) |
| ***MetFrag Relaunched (Eawag EQEx n=289)*** | | | | |
| PubChem 2016 MSMS,RT,Ref,Pat | 236 (81.7%) | NA | 261 (90.3%) | NA |
| PubChemLite tier0 MS,Ref,Pat,FPSum | 266 (92.0%) | 281 (97.2%) | 281 (97.2%) | 8 (2.8%) |
| PubChemLite tier1 MS,Ref,Pat,FPSum | 266 (92.0%) | 279 (96.5%) | 281 (97.2%) | 8 (2.8%) |
| ***MetFrag Relaunched (Eawag EQExPlus n=310)*** | | | | |
| PubChem 2016 MSMS,RT,Ref,Pat | 196 (63.2%) | NA | 233 (75.2%) | NA |
| PubChemLite tier0 MS,Ref,Pat,FPSum | 227 (73.2%) | 266 (85.8%) | 277 (89.4%) | 31 (10.0%) |
| PubChemLite tier1 MS,Ref,Pat,FPSum | 225 (72.6%) | 264 (85.2%) | 277 (89.4%) | 31 (10.0%) |
| ***MetFrag Relaunched (UFZ n=226)*** | | | | |
| PubChem 2016 MSMS,RT,Ref,Pat | 165 (73.0%) | NA | 188 (83.2%) | NA |
| PubChemLite tier0 MS,Ref,Pat,FPSum | 196 (86.7%) | 213 (94.2%) | 222 (98.2%) | 4 (1.8%) |
| PubChemLite tier1 MS,Ref,Pat,FPSum | 194 (85.8%) | 214 (94.7%) | 222 (98.2%) | 4 (1.8%) |

NA: data not available from original files and non-trivial to recreate accurately as, for missing entries, CIDs and preferred tautomeric forms have shifted since 2016.

Table S2: PubChemLite (November 2019 version [1]) and CompTox [6] results for all datasets combined (CASMI 2016 [3] and MetFrag Relaunched [2]) with MS/MS, plus split by ionisation mode (positive, negative) and ignoring entries missing in either database. The parameters are given in the methods. These results are a preliminary version of the final benchmarking set of 977 used (completely de-duplicated over these datasets) and presented in the main manuscript.

|  | Top 1 (%) | Top 2 (%) | Top 5 (%) | Missing (%) |
| --- | --- | --- | --- | --- |
| ***All data (n=1506)*** | | | | |
| PubChemLite tier0 Ref,Pat,FPSum | 1208 (80.2%) | 1342 (89.1%) | 1402 (93.1%) | 89 (5.9%) |
| PubChemLite tier1 Ref,Pat,FPSum | 1203 (79.9%) | 1340 (89.0%) | 1403 (93.2%) | 87 (5.8%) |
| CompTox PCSources,Data,Ref | 1191 (79.1%) | 1337 (88.8%) | 1409 (93.6%) | 71 (4.7%)* |
| ***Negative mode only, excluding missing entries (n=409)*** | | | | |
| PubChemLite tier0 Ref,Pat,FPSum | 351 (85.8%) | 390 (95.4%) | 406 (99.3%) | 0 (0%) |
| PubChemLite tier1 Ref,Pat,FPSum | 351 (85.8%) | 388 (94.9%) | 404 (98.8%) | 0 (0%) |
| CompTox PCSources,Data,Ref | 353 (86.3%) | 393 (96.1%) | 408 (99.8%) | 0 (0%) |
| ***Positive mode only, excluding missing entries (n=948)*** | | | | |
| PubChemLite tier0 Ref,Pat,FPSum | 814 (85.9%) | 898 (94.7%) | 936 (98.7%) | 0 (0%) |
| PubChemLite tier1 Ref,Pat,FPSum | 808 (85.2%) | 896 (94.5%) | 937 (98.8%) | 0 (0%) |
| CompTox PCSources,Data,Ref | 803 (84.7%) | 889 (93.8%) | 993 (98.4%) | 0 (0%) |

*This may be slightly overestimated due to tautomer mismatching in the InChIKey first block due to different structure standardization methods used by the two databases.

## Leveraging Annotation Content

Table S3: Performance of Annotation Scores (no MS/MS) with the AgroChemicals dataset from PubChemLite_tier1 (14 Jan 2020, n=1336) assessed on PubChemLite_tier1 (14 Jan 2020 version) with scoring terms. (a) PMID_Count, Patent_Count and AgroChemInfo (b) PMID_Count, Patent_Count and (c) on the full PubChem database (live query, 22 Jan 2020, 102,404,298 compounds) with scoring terms equivalent to PMID_Count and Patent_Count. Note: the AgroChemInfo term is not available for the full database via the API. This data is included in the main text (Figure 6); script and file on the ECI GitLab pages [7, 8].

| ***Agrochemicals (Additional File 4), n=1336*** |  | Top 1 | Top 2 | Top 5 | Top 10 | Missing |
| --- | --- | --- | --- | --- | --- | --- |
| (a) PubChemLite tier1 (Jan 14, 2020 version): Patents, PubMedRefs, AgroChemScore | N | 1058 | 1244 | 1312 | 1316 | 20* |
|  | % | 79.20% | 93.10% | 98.20% | 98.50% | 1.50% |
| (b) PubChemLite tier1 (Jan 14, 2020 version): Patents, PubMedRefs | N | 940 | 1117 | 1238 | 1282 | 20* |
|  | % | 70.40% | 83.60% | 92.70% | 96.00% | 1.50% |
| (c) Full PubChem Query (Jan 22, 2020): Patents, PubMedRefs | N | 773 | 1005 | 1135 | 1200 | 20* |
|  | % | 57.90% | 75.20% | 84.90% | 89.80% | 1.50% |


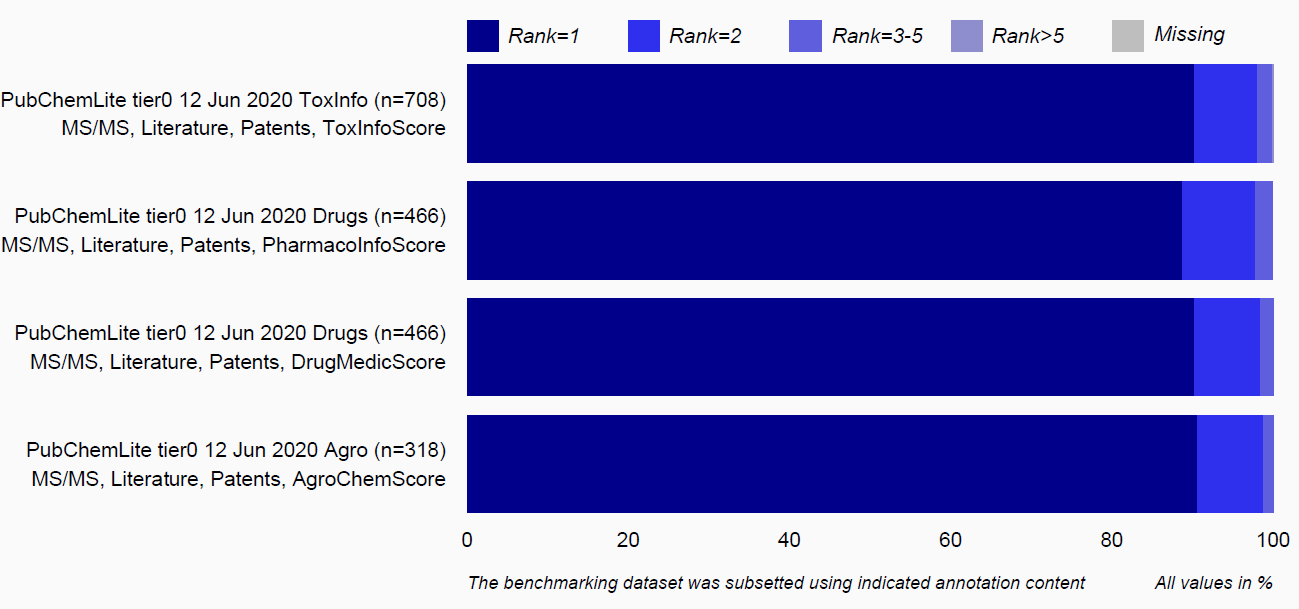


Figure S3: PubChemLite (12 Jun 2020 version) with the benchmarking set (Additional File 2) subset according to annotation category and run in MetFrag with the corresponding annotation scoring term. See main manuscript for more details. Script (plus link to data files) is available from [5]. Figure template from [4].

# Additional Methods

Table S4: Summary of MetFrag-relevant parameters for the various datasets, controlled via scripts available at the ECI GitLab pages [8]. Functions mentioned in column 3 are in the PCLite_eval_support.R script.

| Dataset | Database | Function | Parameters |
| --- | --- | --- | --- |
| All | All | All | ppm=5, mzabs=0.001, frag_ppm=5, useMonaIndiv = TRUE, useMoNAMetFusion = FALSE |
| CASMI | PCLite | runPCLiteEval. CASMI | Charged exact mass from PRECURSOR_MZ column, polarity from ION_MODE column (sets adduct and fragmentation modes) |
| MetFragRL EA | PCLite | runPCLiteEval.EA | Neutral exact mass from NeutralPrecMass column (candidate search), polarity from Mode column (sets mode for fragmentation) |
| MetFragRL EQEx, UF, EQExPlus | PCLite | runPCLiteEval. EQtoUF | Neutral exact mass from NeutralPrecMass column (candidate search), polarity from isPos column (sets mode for fragmentation) |
| PCLite BM | PCLite | runPCLiteEval. BM | Neutral exact mass from ExactMass column (candidate search), polarity from isPos column (sets mode for fragmentation), molecular formula (for testing) from the MolecularFormula column. Additional annotation terms were controlled by “extra_terms”, mapped to headers, added to LocalCSVScoreTerms and weighted with 1 |
| All (unless otherwise specified) | PCLite |  | LocalCSVScoreTerms = PubMed_Count, Patent_Count, FPSum (Nov 2019); LocalCSVScoreTerms = PubMed_Count, Patent_Count, AnnoTypeCount (Jan 2020 and later). LocalCSVScoreWeights = 1, 1, 1 |
| CASMI | CompTox | runCompToxEval. CASMI | Charged exact mass from PRECURSOR_MZ column, polarity from ION_MODE column (sets adduct and fragmentation modes) |
| MetFragRL EA | CompTox | runCompToxEval. EA | Neutral exact mass from NeutralPrecMass column (candidate search), polarity from Mode column (sets mode for fragmentation) |
| MetFragRL EQEx, UF, EQExPlus | CompTox | runCompToxEval. EQtoUF | Neutral exact mass from NeutralPrecMass column (candidate search), polarity from isPos column (sets mode for fragmentation) |
| PCLite BM | CompTox | runCompToxEval. BM | Neutral exact mass from NeutralPrecMass column (candidate search), polarity from isPos column (sets mode for fragmentation) |
| All (unless otherwise specified) | CompTox |  | LocalCSVScoreTerms = NUMBER_OF_PUBMED_ARTICLES, PUBCHEM_DATA_SOURCES, DATA_SOURCES  LocalCSVScoreWeights = 1, 1, 1 |

# List of Abbreviations

| CASMI | Critical Assessment of Small Molecule Identification |
| --- | --- |
| CID | PubChem Compound Identifier |
| CompTox | US EPA CompTox Chemicals Dashboard |
| DTXCID | DSSTox Compound Identifier (from CompTox) |
| DTXSID | DSSTox Substance Identifier (from CompTox) |
| ECI | Environmental Cheminformatics group (at the University of Luxembourg) |
| FPSum | Addition of fingerprint bits to form a scoring term used in PubChemLite |
| IKFB | InChIKey First Block |
| MetFragRL | MetFrag Relaunched |
| MoNA | MassBank of North America |
| MS/MS | Tandem Mass Spectrum, MS2 |
| PCL, PCLite | PubChemLite |
| PMID | PubMed Identifier |

# References

1. Bolton EE, Schymanski EL (2019) PubChemLite tier0 and tier1 (Version 0.1.0) [Data set]. DOI:10.5281/zenodo.3548654

2. Ruttkies C, Schymanski EL, Wolf S, et al (2016) MetFrag relaunched: incorporating strategies beyond in silico fragmentation. Journal of Cheminformatics 8:3. https://doi.org/10.1186/s13321-016-0115-9

3. Schymanski EL, Ruttkies C, Krauss M, et al (2017) Critical Assessment of Small Molecule Identification 2016: automated methods. Journal of Cheminformatics 9:22. https://doi.org/10.1186/s13321-017-0207-1

4. Rahlf T (2014) Datendesign mit R: 100 Visualisierungsbeispiele (Data Design with R: 100 Visualisation Examples), 1st Edition. Open Source Press, Munich, Germany

5. Schymanski, Emma (2020) PubChemLite Evaluation Plotting Script. https://git-r3lab.uni.lu/eci/pubchem/-/raw/master/pubchemlite/R/PCLite_eval_support.R. Accessed 10 Nov 2020.

6. US EPA (2020) CompTox MetFrag Files (EPA FTP Site) - CompTox MetFrag Download Files (FTP). ftp://newftp.epa.gov/COMPTOX/Sustainable_Chemistry_Data/Chemistry_Dashboard/MetFrag_metadata_files/. Accessed 10 Nov 2020.

7. Schymanski, Emma (2020) Environmental Cheminformatics GitLab Pages: PubChemLite Figures Folder. https://git-r3lab.uni.lu/eci/pubchem/-/tree/master/pubchemlite/R/figures/. Accessed 27 Oct 2020

8. Schymanski, Emma (2020) Environmental Cheminformatics GitLab Pages: PubChemLite R Script Folder. https://git-r3lab.uni.lu/eci/pubchem/-/tree/master/pubchemlite/R/. Accessed 27 Oct 2020
